# Efficacy and durability of GB10, a novel bispecific VEGF/Ang-2 neutralizing fusion protein, in ocular neovascularization models

**DOI:** 10.3389/fphar.2026.1974368

**Published:** 2026-10-20

**Authors:** Meiqi Hao, Qiongying You, Xiling Wei, Suofu Qin

**Affiliations:** Drug Discovery, Center for Research and Development, Kexing Biopharm Co., Ltd. Shenzhen, Guangdong, China

**Keywords:** GB10, bispecific neutralizing fusion protein, CNV, RNV, durability, efficacy, DL-alpha-aminoadipic acid

## Abstract

**Purpose:**

To evaluate the preclinical efficacy, durability, and ocular biodistribution profiling of GB10, a novel bispecific vascular endothelial growth factor/angiopoietin 2 neutralizing fusion protein, compared with faricimab in models of choroidal and retinal neovascularization.

**Methods:**

Cynomolgus monkeys (cyno) with laser-induced choroidal neovascularization (CNV) received intravitreal GB10 (0.07, 0.7, or 7 mg/eye), faricimab (6 mg/eye), or vehicle. Dutch belted rabbits with DL-alpha-aminoadipic acid-induced retinal neovascularization (RNV) received GB10, faricimab, or vehicle. Chinchilla rabbits were used for biodistribution. Efficacy and durability were assessed by fluorescein fundus angiography in cyno CNV model and rabbit RNV model. Tissue concentrations were quantified by ELISA.

**Results:**

In the CNV model, GB10 at 0.7 mg/eye achieved similar anti-leakage inhibition at a 9-fold lower dose relative to faricimab at 6 mg/eye (grade 4 incidence: 54.17% vs. 76.39% at week 1). By week 2, GB10 (0.7 and 7 mg/eye) achieved 0% incidence of grade 4 leakage, sustained through week 4. In the RNV model, GB10 sustained inhibitory activity for ≥12 weeks, whereas faricimab showed effects up to 4 weeks for FFA score and 8 weeks for grade 4 leakage. GB10 demonstrated high distribution in retina/choroid (≈35% of total exposure) and minimal systemic exposure (serum proportion 0.02%).

**Conclusion:**

In preclinical ocular neovascularization models, GB10 shows desirable efficacy at a 9-fold lower dose versus faricimab in cyno CNV model, alongside extended durability of ≥12 weeks (faricimab: 4 weeks for FFA score and 8 weeks for grade 4 leakage area) in rabbit RNV model. These preclinical findings demonstrate GB10’s encouraging prospects as a next-generation therapeutic candidate, with efficacy profiles to be confirmed in clinical investigations.

## 1 Introduction

Neovascular and exudative eye diseases, including age-related macular degeneration (AMD), diabetic macular edema (DME), diabetic retinopathy (DR), and retinal vein occlusion (RVO), represent leading causes of substantial visual loss and blindness worldwide (Bourne et al., 2013). Among these ocular neovascular disease, neovascular age-related macular degeneration (nAMD) is the primary driver of severe visual decline. This progressive pathology is initiated by retinal pigment epithelium dysfunction, drusen accumulation, and choriocapillaris hypoperfusion, which disrupt Bruch’s membrane and foster a hypoxic, pro-angiogenic microenvironment, ultimately leading to the development of retinal and choroidal neovascular diseases (Thomas et al., 2021; Sheth et al., 2025; Wang et al., 2025).

The development of intravitreal (IVT) anti-vascular endothelial growth factor (VEGF) agents, such as ranibizumab, aflibercept, and brolucizumab, revolutionized the management of nAMD, enabling vision stabilization or improvement in the patients (Heier et al., 2012; Dugel et al., 2020). However, a major clinical limitation persists: the shorter duration of these drugs necessitate frequent injections (monthly or bimonthly) to maintain efficacy, imposing a substantial burden on patients, caregivers, and healthcare systems (Xu et al., 2022). Poor treatment adherence due to their high-frequency dosing is a key contributor to suboptimal real-world visual outcomes (Holz et al., 2015). Several long-acting anti-angiogenic therapeutics have since entered clinical practice to address this dosing burden. For enhanced efficacy, one strategy is that 8 mg high-dose aflibercept has been approved with three initial 4-weekly loading dose followed by maintenance every 8–16 weeks (Wykoff et al., 2023); the other is for dual-target therapy, faricimab that inhibits VEGFA/Ang2 is approved for four initial 4-weekly loading doses with subsequent dosing intervals guided by optical coherence tomography and visual acuity assessments at weeks 8 and 12 (Fulcher and Ghanchi, 2026). While these agents have successfully prolonged treatment intervals relative to monthly therapy, the long-acting regimens are still required for patients with persistent leakage. Consequently, there is an urgent, unmet need for novel therapeutic strategies with extended durability that can reduce injection frequency without compromising efficacy.

A promising strategy to address this need is the development of multi-targeting agents that simultaneously inhibit complementary pathways driving ocular neovascularization pathogenesis, potentially achieving more profound and sustained suppression of neovascularization. Beyond VEGF, angiopoietin 2 (Ang-2), a critical context-dependent cytokine in the Ang/Tie2 signaling pathway, is upregulated at sites of active neovascular remodeling and inflammation. By competitively binding to the Tie2 receptor against Ang-1, Ang-2 promotes endothelial cell activation, disrupts neovascular stability, increases permeability, and facilitates inflammatory cell infiltration (Fiedler and Augustin, 2006; Eklund and Saharinen, 2013; Akwii et al., 2019). Within the hypoxic and inflammatory microenvironment of ocular neovascularization, VEGF initiates neovascular sprouting, while Ang-2 supports the subsequent destabilization and pathological maturation of nascent vessels (Campochiaro, 2015; Wolf and Langmann, 2019). Overactivation of both VEGF and Ang-2 signaling pathways collectively induces abnormal neovascular proliferation and leakage, which synergistically drives pathological angiogenesis and renders co-inhibition a rational therapeutic approach.

Building upon this rationale, we engineered GB10, a novel neutralizing fusion protein consisting of VEGF-Trap and an non-humanized anti-Ang-2 variable heavy domain of heavy-chain (VHH) antibody of camelid origin to simultaneously block the pathways of VEGF family and Ang-2 for enhanced and sustained efficacy (Wei et al., 2025). GB10 differs markedly from faricimab with respect to molecular topology and target inhibitory spectrum. Faricimab adopts a conventional 1 + 1 bivalent bispecific antibody format constructed from separate anti-VEGF-A and anti-Ang-2 Fab fragments, which only neutralizes VEGF-A and Ang-2. In contrast, GB10 features a symmetric tetravalent 2 + 2 architecture incorporating a VEGF-Trap module capable of concurrent neutralization of VEGF-A, VEGF-B, and placental growth factor (PlGF). This distinct structural configuration confers improved ligand-neutralizing potency in biochemical assays: the IC_50_ values of GB10 for VEGF and Ang-2 inhibition are 7.5-fold and 94-fold lower than those of faricimab, respectively (Wei et al., 2025).

Laser-induced CNV and chemical toxin-induced RNV are widely used preclinical models for ocular neovascular diseases. While the CNV model recapitulates neovascularization triggered by Bruch’s membrane rupture, spontaneous lesion regression restricts its application for long-term efficacy test. In comparison, toxin-induced RNV produces persistent neovascular leakage ideal for long durability assessment (Grossniklaus et al., 2010; Fabian-Jessing et al., 2022). To evaluate whether GB10 can last for longer duration in inhibiting neovascularization due to its tetravalent design and multitargeting, we adopted a DL-alpha-aminoadipic acid (DL-AAA)-induced rabbit RNV model. This model is developed based on the pivotal role of retinal Müller cells in blood-retinal barrier (BRB) maintenance. DL-AAA, a selective Müller cell gliotoxin (Olney, 1982), induces Müller cell injury and subsequent BRB disruption, a process characterized by the downregulation of tight junction proteins and sustained upregulation of VEGF (Shen et al., 2010). These pathological events drive the formation of stable retinal neovascularization and neovascular leakage that persist for several months.

Taking these two ocular models, we have demonstrated that GB10 showed sustained neovascular leakage-inhibitory activity for more than 12 weeks and rapid low-dose inhibition, though short of statistical significance between GB10 and faricimab. Biodistribution analysis reveals minimal systemic exposure and preferential target-tissue accumulation. These findings highlight GB10’s promising potential for ocular neovascular diseases.

## 2 Materials and methods

### 2.1 Test agents

GB10 was provided by Kexing Biopharm Co., Ltd., and its detailed characteristics have been reported previously (Wei et al., 2025). Faricimab was obtained from F. Hoffmann-La Roche AG, with batch number B1535B01.

### 2.2 Ethical approval

All animal procedures were conducted at InnoStar Bio-tech Nantong Co. Ltd., in compliance with the Institutional Animal Care and Use Committee (IACUC) guidelines. The study adhered to relevant animal welfare regulations, including the U.S. National Research Council’s Guide for the Care and Use of Laboratory Animals (2011), China’s Regulation on the Administration of Laboratory Animals (2017 revision), and the Jiangsu Measures for the Administration of Laboratory Animals (2008). The protocols were approved by the IACUC under the following ethics approval numbers: IACUC-2024-M-1011, IACUC-2025-R-049 and IACUC-2024-R-963.

### 2.3 Laser-induced CNV in cyno monkeys

#### 2.3.1 Animals

A total of 20 male cyno monkeys (body weight 2.9–7.4 kg), supplied by Huazheng Laboratory Animal Breeding Centre and Guangxi Weimei Bio-tech Co., Ltd., were used in this study. Male animals were selected in accordance with standard practice for laser-induced CNV non-human primate models (Isumi et al., 2020; Cheong et al., 2021), to avoid cyclic estrogen fluctuations in females that may increase variability in CNV lesion formation and leakage (Espinosa-Heidmann et al., 2005), However, it should be noted that the use of only male animals may limit the external validity of the findings. During the acclimation and observation period, animals were kept in single-housing for husbandry care. Environmental parameters within the animal room were continuously recorded throughout the study, and no other animal species were co-housed in the same room.

#### 2.3.2 Experimental CNV model and treatment protocol

Prior to all ophthalmic examinations and surgical procedures, each animal was anesthetized via intramuscular injection of 5 mg/kg Zoletil (Virbac Laboratories, Batch Number: BN968AA) with 0.5 mg/kg xylazine (Dunhua Shengda Animal Pharmaceutical Co., Ltd., Batch Number: 20240605). For mydriasis, two drops of tropicamide eye drop were applied per application before ophthalmic assessments (Jinyao Yongguang Pharmaceutical Co., Ltd., Hrbei, Batch no: 0042345).

CNV was induced bilaterally in cyno monkeys by laser photocoagulation (0.5 W, 50 μm spot, 0.1 s) by frequency-doubled Nd: YAG laser (Quantel Medical, VITRA) at 532 nm wavelength. Successful lesion formation was defined by Bruch’s membrane rupture, confirmed by immediate bubble formation and optical coherence tomography. Owing to the risk of intra-ocular inflammation following IVT manipulation, animals received 3 consecutive days of post-procedural care, including tobramycin dexamethasone eye drops (Chengdu Qingshan Likang Pharmaceutical Co., Ltd., Batch: J231144), ofloxacin eye ointment (Shenyang Xingqi Pharmaceutical Co., Ltd., Batch: C210201), levofloxacin eye drops (Santen Pharmaceutical (China) Co., Ltd., Batch: CV2209), and daily clinical monitoring was performed by veterinary staff.

D14 after laser injury, eyes were screened via fluorescein fundus angiography (FFA); only those exhibiting at least one grade 4 leakage spot were included in the study, a criterion that ensured clinical relevance (Goody et al., 2011). Following randomization, twenty animals were allocated into five groups (4 animals, 8 eyes per group) to receive vehicle, GB10 (0.07, 0.7, or 7 mg/eye), or faricimab (6 mg/eye). All treatments were administered as a single 50 μL IVT injection per eye on day 14, which was consistent with clinical administration volumes.

#### 2.3.3 The criteria of FFA

Efficacy was evaluated based on FFA outcomes obtained at weeks 1, 2, and 4 post dosing. FFA images were captured using the Microclear Apollo Confocal Retina Ophthalmoscope and analyzed via Heidelberg Eye Explorer software; the leakage area was quantified by manually tracing hyperfluorescent regions within the vitreous on FFA frames. The CNV lesions were scored with a standardized scale as follows: Grade 1: no high fluorescence in the spot, Grade 2: the spot is hyperfluorescent without fluorescein leakage, Grade 3: the spot is hyperfluorescent with mild fluorescein leakage, but leakage does not exceed the border of the spot, Grade 4: the spot is hyperfluorescent with significant fluorescein leakage beyond the boundaries of the spot. The statistical analysis focused on the incidence of grade 4 spots calculated for each eye as follow (Li et al., 2019; Cai et al., 2025):

$$\text{Incidence }\left(\%\right)=\frac{\text{Number}\;\text{of}\;\text{grade}\;4\;\text{spots}}{\text{Number}\;\text{of}\;\text{laser}‐\text{induced}\;\text{lesions}\;}\times 100\%$$

#### 2.3.4 Animal randomization

Animals with successful CNV induction in both eyes were selected for grouping with Block randomization, stratified by the total number of grade 4 laser spots after CNV development. The block size was set to 4. Within each block animals were randomly assigned to the five treatment groups to balance baseline lesion severity across groups.

#### 2.3.5 FFA image analysis

Cross-assessor arbitration procedures were deployed to mitigate subjective measurement bias. Briefly, the mean value from the two independent assessors was adopted when the measurement error was less than 4% following the predefined assessment criteria. A third senior assessor will perform supplementary delineation for cases with error exceeding 4% and the final measurement was calculated as the arithmetic average of three independent evaluations to reduce measurement deviation.

### 2.4 DL-AAA-induced RNV in rabbits

#### 2.4.1 Animals

Dutch belted rabbits (body weight: 1.5–5 kg; age: 7–8 months) were obtained from Pizhou Dongfang Breeding Co. Ltd. and acclimatized for at least 1 week prior to experimentation. Before any ophthalmic procedure, animals received topical levofloxacin for antimicrobial prophylaxis and compound tropicamide for pupillary dilation. Anesthesia was induced via intramuscular injection of ketamine (40 mg/kg) (Manufacturer: Jiangsu Pocon Pharmaceutical Industries Co., Ltd., Batch Number: 20241204). and xylazine (5 mg/kg) (Manufacturer: Dunhua Shengda Animal Pharmaceutical Co., Ltd., Batch Number: 20240605). During procedures, the cornea was kept moist with topical 0.9% sodium chloride. Postoperative care included topical administration of levofloxacin (Manufacturer: Santen Pharaceutical Co., Ltd., Noto Plant, Batch Number: SCV00057) to manage potential inflammation.

#### 2.4.2 DL-AAA preparation, RNV induction, and model qualification

A stock solution of DL-AAA was prepared by dissolving 128.93 mg of DL-AAA (Manufacturer: Sigma-Aldrich (Shanghai) Trading Co. Ltd., Batch Number: BCCH2077) in 1 mL of 1 M HCl. This was diluted with 4 mL of 0.9% sterile saline, and the pH was adjusted to 7.4 ± 0.5 with 1 M NaOH. The final solution was filtered through a 0.22 μm filter immediately before use. Following baseline ophthalmic examinations, RNV was induced by a single bilateral IVT injection of 90 μL of 80 mM DL-AAA in dutch belted rabbits. Disease progression was monitored via follow-up examinations for up to 24 weeks. Examinations included slit-lamp microscopy, fundus photography (FP), and FFA. For FFA, 20% sodium fluorescein (0.1 mL/kg) (Manufacturer: Guangzhou Baiyunshan Mingxing Pharmaceutical Co., Ltd., Batch Number: 1242601) was injected intravenously. Images were acquired with a Heidelberg Retina Angiograph (HRA) system and analyzed using Heidelberg Eye Explorer software. The leakage area was quantified by tracing the hyperfluorescent region in the vitreous.

#### 2.4.3 Drug administration and efficacy evaluation

Once stable RNV was established, eyes received a single IVT injection (50 μL/eye, n = 5) of GB10 (140 mg/mL), faricimab (120 mg/mL, n = 5), or GB10 placebo (vehicle, n = 5). Modeling and efficacy evaluation were achieved by FFA at designated time points. FFA grading was performed as previously described (Kumar et al., 2022). FFA images comprised of central, nasal and temporal retinal vasculature were aligned and 4 grids were placed on temporal (R1, R2, R3, and R4) and nasal (R5, R6, R7, and R8) retinal vasculature areas, as show in Figure 1A. At every observational time point, the severity of neovascular leakage was assessed as follows: Grade 0: major vessels straight some tortuosity of smaller vessels. no vessel dilation, Grade 1: increased tortuosity or neovessels with no or very slight leakage and clear background, Grade 2: increased tortuosity or neovessels with slight leakage and clear background, Grade 3: moderate leakage from all or some neovascular vessels with visible background, Grade 4: severe leakage from all neovessels with poorly/not visible background, the detailed criteria of which are illustrated in Figure 1B. The percentage change in FFA score from baseline was calculated as follow:

$$\text{FFA score }\left(\%\right)=\frac{\text{Post}‐\text{treatment}\;\text{score}}{\text{Post}‐\text{treatment}\;\text{score}\;}\times 100\%$$

**FIGURE 1:**
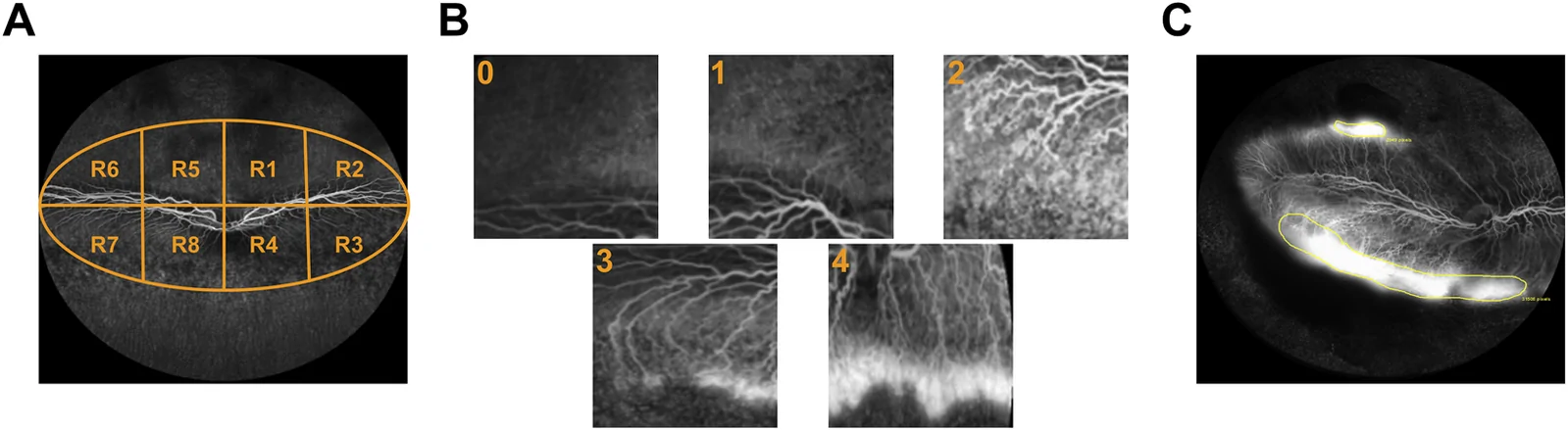
FFA grading system and schematic diagram of grade 4 leakage area for DL-AAA induced rabbit RNV model. **(A)** Schematic diagram of the fundus divided into 8 regions (R1, R2, R3, R4, R5, R6, R7, R8). **(B)** FFA scores grading of DL-AAA rabbit eye. The numbers 0–4 represent the FFA scores of grade 0–4, as described in the Materials and Methods (DL-AAA preparation, RNV induction, and model qualification). **(C)** Schematic diagram of grade 4 leakage area, the yellow outline represents the grade 4 leakage range.

Since grade 4 leakage is associated with pathological neovascular leakage observed in patients, we evaluated therapeutic efficacy by quantifying the reduction in grade 4 leakage area. FFA images were captured using the Heidelberg Retina Angiograph system and analyzed via Heidelberg Eye Explorer software; the leakage area was quantified by manually tracing hyperfluorescent regions within the vitreous on FFA frames (Figure 1C). The mean of the two delineations was used for subsequent analyses. The percentage change from baseline was calculated as follow:

$$\text{Grade }4\text{ leakage area }\left(\%\right)=\frac{\text{Leakage}\;\text{area}\;\text{of}\;\text{time}\;\text{point}}{\;\text{Leakage}\;\text{area}\;\text{of}\;\text{baseline}}\times 100\%$$

#### 2.4.4 Animal randomization

Eyes with valid lesion induction were screened based on ophthalmic imaging findings acquired at the final examination prior to group assignment. Subjects were stratified into distinct blocks according to the number of grade 4 leakage lesions per eye, followed by sample allocation via stratified block randomization. Within each block containing eyes with comparable lesion burden, random sequences generated by statistical software evenly distributed the specimens across the three treatment groups. This stratified block design, stratified by baseline lesion load, inherently limits intergroup divergence in baseline lesion severity and achieves approximate balance of lesion profiles across cohorts, consistent with standard pragmatic laboratory practices for small-scale exploratory preclinical ocular studies.

#### 2.4.5 FFA image analysis

FFA image grading and leakage-area quantification were performed following the same image analysis and inter-assessor arbitration procedure described in the section of 2.3.

### 2.5 The PK profile and ocular distribution

Cross sectional sacrificial sampling was deployed for PK profile and ocular distribution. A total of 48 chinchilla rabbits (24 males, 24 females) received a single bilateral IVT injection of GB10 (7 mg/eye, 50 μL). Animals were fasted overnight pre-dose and randomized into eight terminal sampling time points (4 h, 24 h, 72 h, 168 h, 336 h, 504 h, 672 h, and 1,008 h post-dose; n = 6 animals per group, sex-balanced). Following serum collection, animals were euthanized via exsanguination under deep anesthesia induced by intramuscular ketamine (40 mg/kg) combined with xylazine (5 mg/kg); ocular tissues (vitreous/aqueous humor, cornea, lens, retina, choroid, opticnerve, conjunctiva) were collected at designated times. Quantification of GB10 concentrations in serum and ocular tissues was performed via a fully validated ELISA assay. This immunoassay employs immobilized recombinant human VEGF_165_ as the capture reagent and human angiopoietin-2 as detection reagent to quantify free GB10 rather than total GB10. Pharmacokinetic parameters were derived via non-compartmental analysis (Phoenix WinNonlin 8.3) in accordance with established guidelines for non-compartmental pharmacokinetics.

### 2.6 Statistical analysis

For cyno CNV, measurements from the two eyes of each animal were averaged prior to statistical analysis. For both cyno CNV and rabbit RNV models, all analyses were conducted versus the vehicle. Normality was assessed using the Shapiro-Wilk test (*p* > 0.05 required for parametric analysis). Data meeting normality assumptions were analyzed with one-way ANOVA. Otherwise, non-parametric equivalents were employed. Statistical significance was defined as *p* < 0.05 (two-tailed). Due to the small sample size, mixed models was not adopted for statistical analysis here.

## 3 Results

### 3.1 GB10 achieved a rapid therapeutic effect in cyno CNV model

GB10 exhibited better efficacy in a laser-induced cyno CNV model in our pilot study (Wei et al., 2025), we further investigated the efficacy of GB10 at varying doses in the same model to corroborate our previous findings. To improve statistical reliability, the sample size was increased to 4 animals per group, and the efficacy of GB10 was compared with that of faricimab at its clinically approved dose of 6 mg/eye (50 μL). Figure 2A illustrated the CNV induction procedure, IVT injection and post-dosing imaging schedule.

**FIGURE 2:**
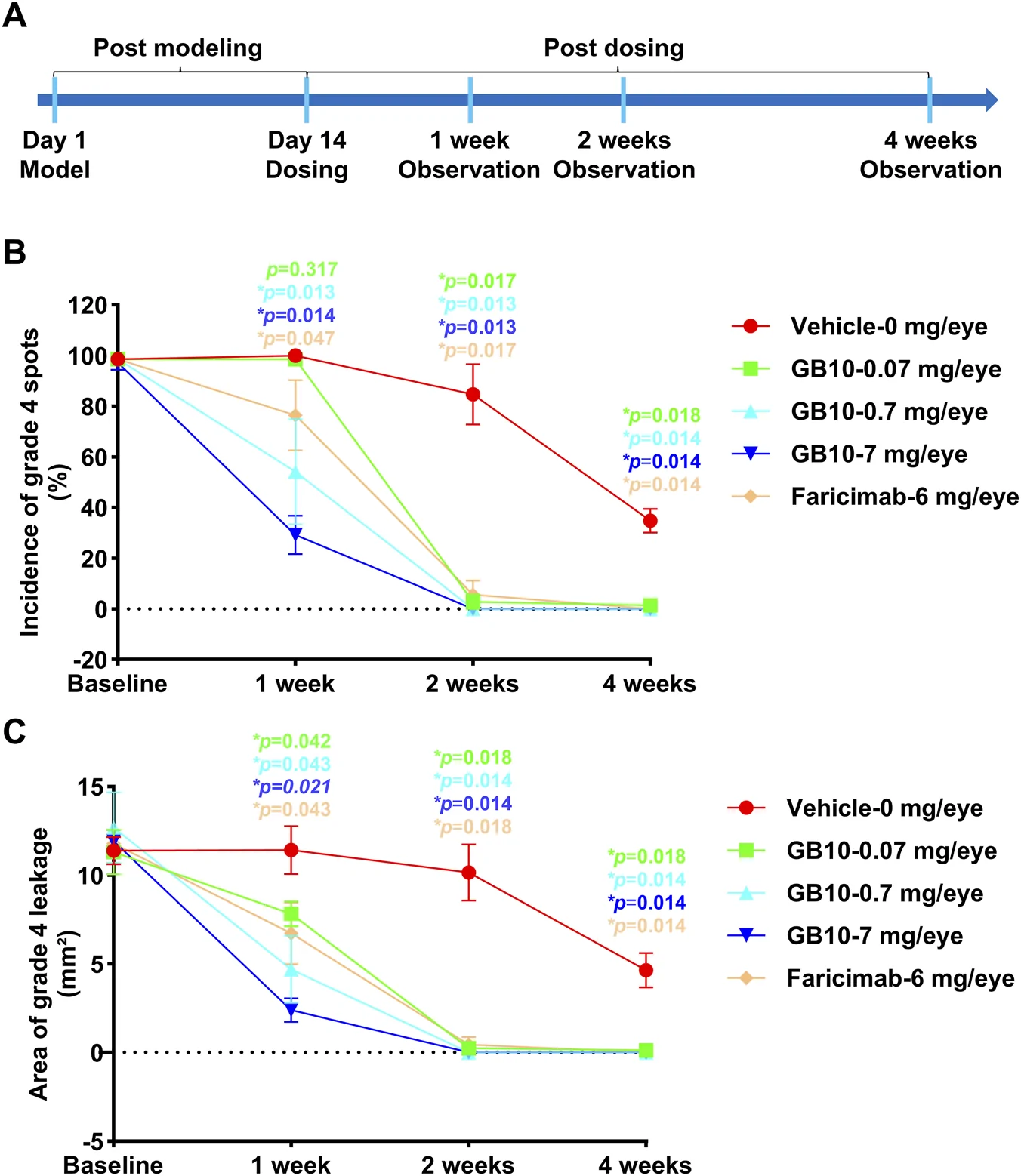
Efficacy of GB10 in cyno CNV model. **(A)** Schematic representation of the experimental setup including modeling, grouping/dosing, and efficacy readouts. **(B,C)** The incidence (%) and area (mm^2^) of grade 4 leakage on baseline, 1, 2, and 4 weeks after IVT of vehicle, GB10 (0.07, 0.7 and 7 mg/eye) and faricimab (6 mg/eye), respectively. The data were presented as Mean ± SEM; Compared with vehicle,*p < 0.05, **p < 0.01, ***p < 0.001. n = 4 animals per group (8 eyes in total), for each animal with bilateral eyes, values from left and right eyes were averaged, and the animal-level mean was used for statistical analysis.

In the vehicle, robust neovascular leakage with early fluorescein leakage was observed at photocoagulation sites, progressing to confluent sheets of late-phase hyperfluorescence. GB10 treatment resulted in a dose dependent reduction in the incidence and leakage area of grade 4 leakage. At 1 week post-dosing, the incidences were 98.61%, 54.17%, and 29.16% for 0.07, 0.7, and 7 mg/eye GB10, versus 100% in the vehicle and 76.39% in the faricimab (Figure 2B; Table 1); corresponding grade 4 leakage areas were 7.82, 4.69, and 2.39 mm^2^, compared with 11.43 mm^2^ in the vehicle and 6.73 mm^2^ in the faricimab (Figure 2C; Table 2). Pairwise comparisons of grade 4 incidence at 1 week post-dosing (Table 3) revealed a statistically significant difference between GB10 0.07 mg/eye and 0.7 mg/eye (*p* = 0.017), demonstrating a significant dose-effect relationship for GB10. The comparison between GB10 7 mg/eye and faricimab 6 mg/eye approached the threshold for statistical significance (*p* = 0.059). These findings indicated numerical trend towards earlier onset of action for GB10 at doses of 0.7 and 7 mg/eye relative to that for faricimab. By 2 weeks post-dosing, GB10 at 0.7 and 7 mg/eye achieved sustained inhibitory activity (0% incidence and area of grade 4 leakage), and all active treatment groups showed significant suppression compared with vehicle, an effect sustained through 4 weeks post-dosing. These findings revealed a trend toward more rapid inhibition of neovascular leakage by GB10 than that by faricimab, with GB10 at a 9-fold lower dose (the similar efficacy was attained at 0.7 mg/eye for GB10 versus 6.0 mg/eye for faricimab, representing a 9-fold lower dose difference between the two agents).

**TABLE 1:** The incidence of grade 4 spots (%) in cyno CNV model.

Group	Baseline	1 week post-dosing	2 weeks post-dosing	4 weeks post-dosing
Vehicle-0 mg/eye	98.61 ± 1.39	100.00 ± 0.00	84.72 ± 11.87	34.72 ± 4.74
GB10-0.07 mg/eye	98.61 ± 1.39	98.61 ± 1.39	2.78 ± 2.78*	1.39 ± 1.39*
GB10-0.7 mg/eye	98.61 ± 1.39	54.17 ± 20.83*	0.00 ± 0.00*	0.00 ± 0.00*
GB10-7 mg/eye	97.22 ± 2.78	29.16 ± 7.65*	0.00 ± 0.00*	0.00 ± 0.00*
Faricimab-6 mg/eye	98.61 ± 1.39	76.39 ± 13.87*	5.56 ± 5.56*	0.00 ± 0.00*

Data were presented as Mean ± SEM; Compared with vehicle, *p < 0.05, **p < 0.01, ***p < 0.001. n = 4 animals per group (8 eyes in total), for each animal with bilateral eyes, values from left and right eyes were averaged, and the animal-level mean was used for statistical analysis.

**TABLE 2:** The area of grade 4 leakage (mm^2^) in cyno CNV model.

Group	Baseline	1 week post-dosing	2 weeks post-dosing	4 weeks post-dosing
Vehicle-0 mg/eye	11.39 ± 0.78	11.43 ± 1.36	10.16 ± 1.59	4.64 ± 0.96
GB10-0.07 mg/eye	11.30 ± 1.26	7.82 ± 0.70*	0.23 ± 0.23*	0.12 ± 0.12*
GB10-0.7 mg/eye	12.69 ± 1.98	4.69 ± 1.91*	0.00 ± 0.00*	0.00 ± 0.00*
GB10-7 mg/eye	11.92 ± 0.65	2.39 ± 0.67*	0.00 ± 0.00*	0.00 ± 0.00*
Faricimab-6 mg/eye	11.80 ± 0.39	6.73 ± 1.73*	0.44 ± 0.44*	0.00 ± 0.00*

Data were presented as Mean ± SEM; Compared with vehicle, *p < 0.05, **p < 0.01, ***p < 0.001. n = 4 animals per group (8 eyes in total), for each animal with bilateral eyes, values from left and right eyes were averaged, and the animal-level mean was used for statistical analysis.

**TABLE 3:** Pairwise comparison of grade 4 incidence in cyno CNV model.

Pairwise comparison	P Value
GB10-0.07mg/eye vs. 0.7mg/eye	0.017*
GB10-0.7mg/eye vs. 7mg/eye	0.056
GB10-7mg/eye vs. faricimab	0.059

*p < 0.05. The above P-values were for the 1-week post-dosing.

### 3.2 DL-AAA induced RNV demonstrated stable leakage for up to 24 weeks

A rabbit RNV model in which neovascular leakage could last for over 6 months has been developed to evaluate the duration of efficacy. Before modeling, the normal retinal features in both FP and FFA were visualized, including the medullary rays, optic nerve head (ONH), and retinal vasculature. Neovascularization was observed as early as 2 weeks (data not shown) after the DL-AAA injection as evidenced by engorged retinal vessels growing into the visual streak area from the vasculature of the optical disc (Figure 3A, top panel). The FFA of the neovascular leakage were characterized by well-demarcated boundaries discernible at an early phase of angiography and progressive leakage in later phase of angiography. The hyperfluorescent leaking lesion increased rapidly in the first 2 weeks (data not shown), relatively stabilized within 4–8 weeks, and was persistent throughout the entire observation of 24 weeks without regression (Figure 3A, lower panel). No abnormalities affecting the efficacy evaluation were observed via slit-lamp microscopy, as illustrated in Figure 3B.

**FIGURE 3:**
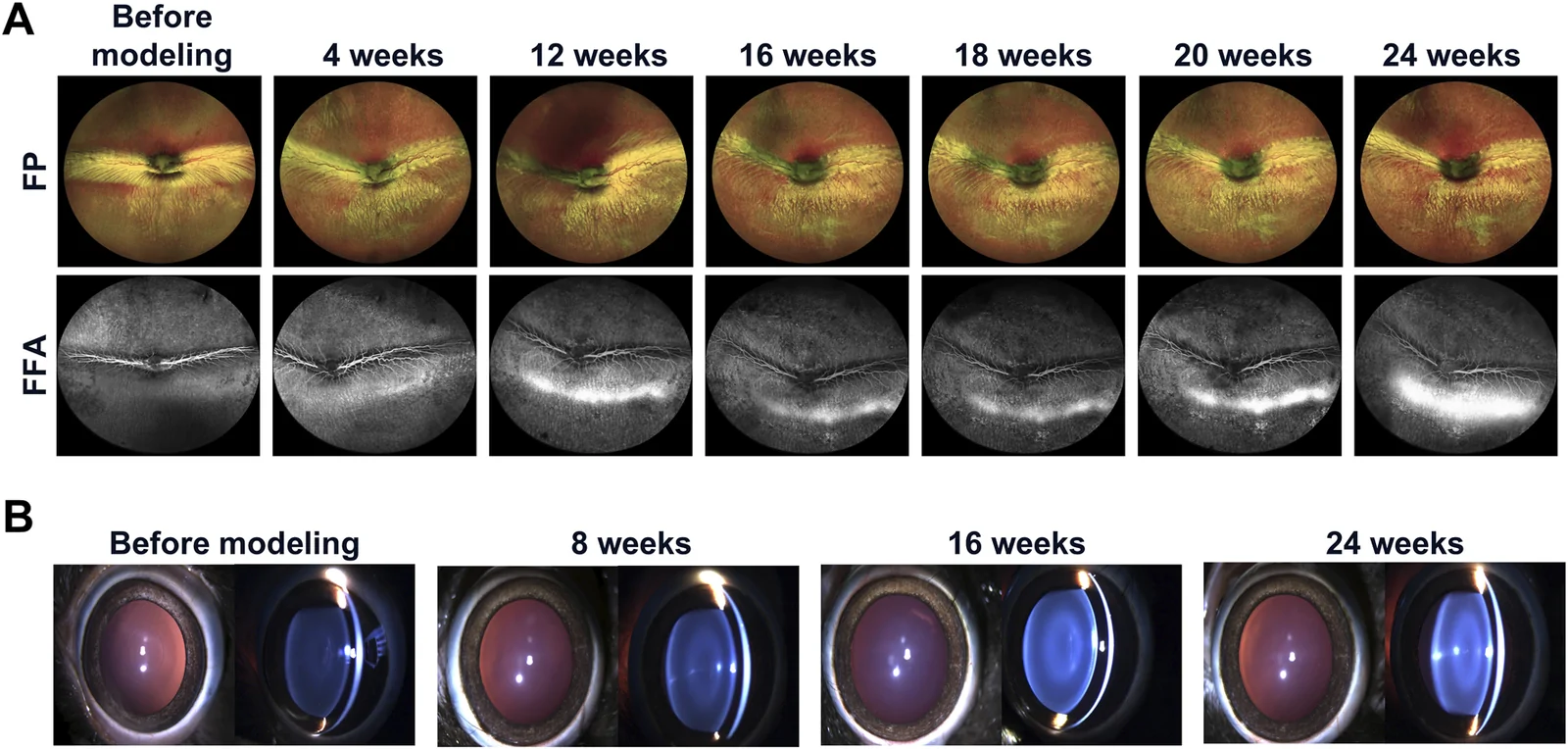
RNV development after single DL-AAA IVT in dutch belted rabbits. **(A)** FP (top panel) and FFA (lower panel) were taken from a representative RNV eye for over 24 weeks. Before chemical toxin-inducing, FP and FFA revealed a normal retinal profile, including the medullary rays, ONH, and retinal vasculature. The process of new blood vessel formation from existing vessel networks and hyperfluorescent areas in the visual streak of the retina was shown from 4 to 24 weeks after DL-AAA injection. **(B)** During the acclimation period, general ophthalmic examinations in the RNV model revealed no significant abnormalities in any animals.

### 3.3 GB10 exhibited sustained inhibitory activity against retinal neovascular leakage in the rabbit RNV model

Following a 3-month induction period with DL-AAA, animals were randomized to receive a single IVT injection of GB10, faricimab, or vehicle. Serial FFA was performed biweekly for 12 weeks post-dosing to assess therapeutic efficacy (Figure 4A). FFA analysis revealed divergent durability profiles between GB10 and faricimab within this animal model. As shown in Figure 4B, minimal leakage recurred in the faricimab group by week 6 post-dosing, progressing to severe leakage by week 12 post-dosing. In contrast, GB10 treatment inhibited RNV and delayed the recurrence of leakage until week 10 post dosing, showing a numerically extended window of inhibitory activity in this model.

**FIGURE 4:**
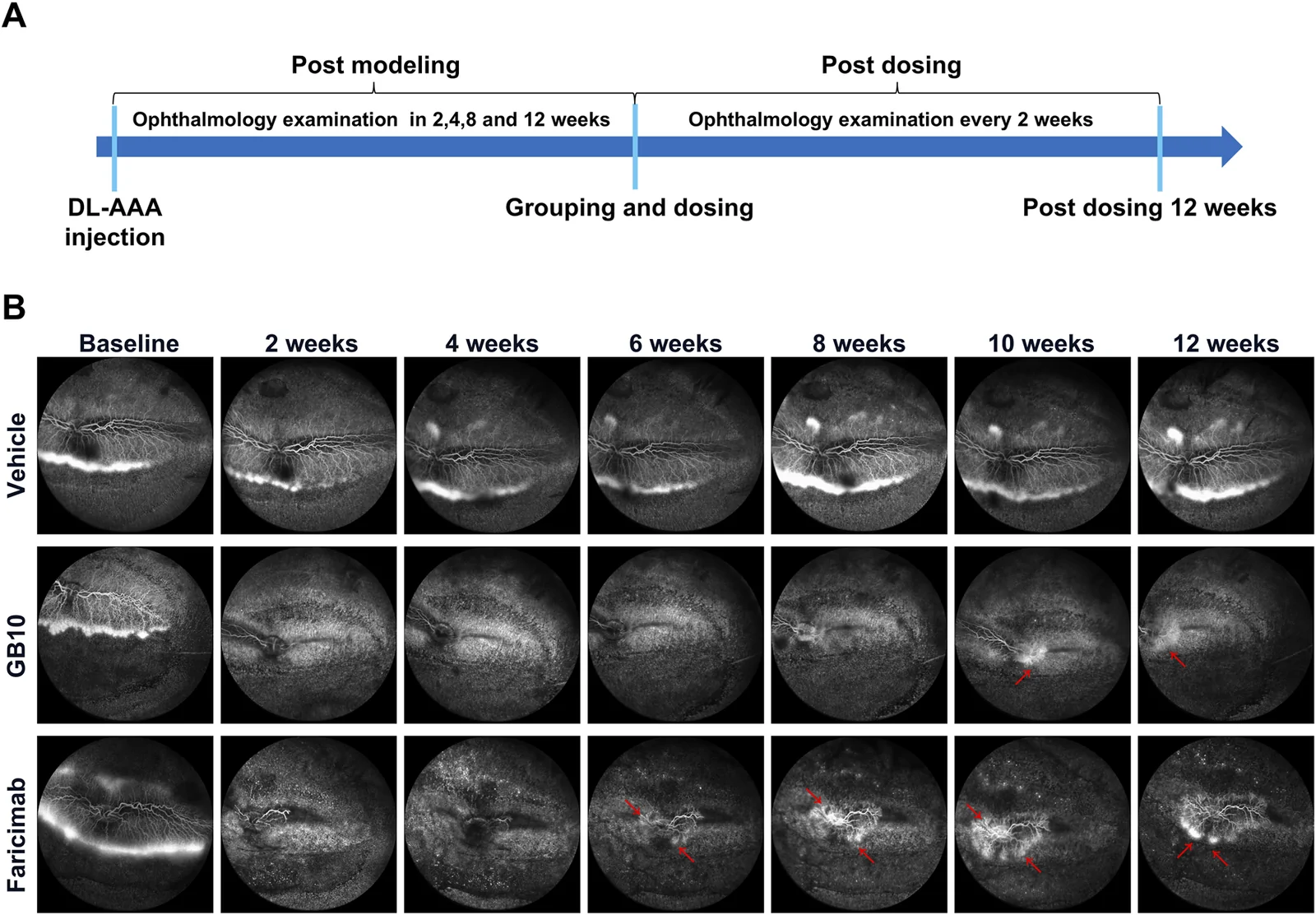
Efficacy of GB10 in a rabbit RNV model. **(A)** Schematic representation of the experimental setup including modeling, grouping, dosing, and efficacy readouts of GB10. **(B)** Representative pictures of FFA on baseline (before dosing) and 2 weeks–12 weeks after IVT of vehicle, GB10 and faricimab, respectively. Red arrows denoted retinal vascular leakage.

The percentage of FFA score at 2, 4, 6, 8, 10, and 12 weeks after administration compared with that before administration were detailed in Figure 5A and Table 4. In the vehicle, there was no significant change in the percentage of scores in each period compared with that before administration, and the model was stable for evaluating the pharmacodynamic effects of the test article. Compared to the vehicle, GB10 showed improvements at all time points, and persistently inhibited the leakage through 12 weeks post-dosing. In contrast, faricimab displayed apparent lesion attenuation relative to vehicle only at weeks 2–4 post-dosing, with minimal distinguishable effects noted from week 6 onward. Statistical comparison between GB10 and faricimab (Figure 5A, top black letter) showed increased difference with expended observation time, however, failed to demonstrated statistical significance.

**FIGURE 5:**
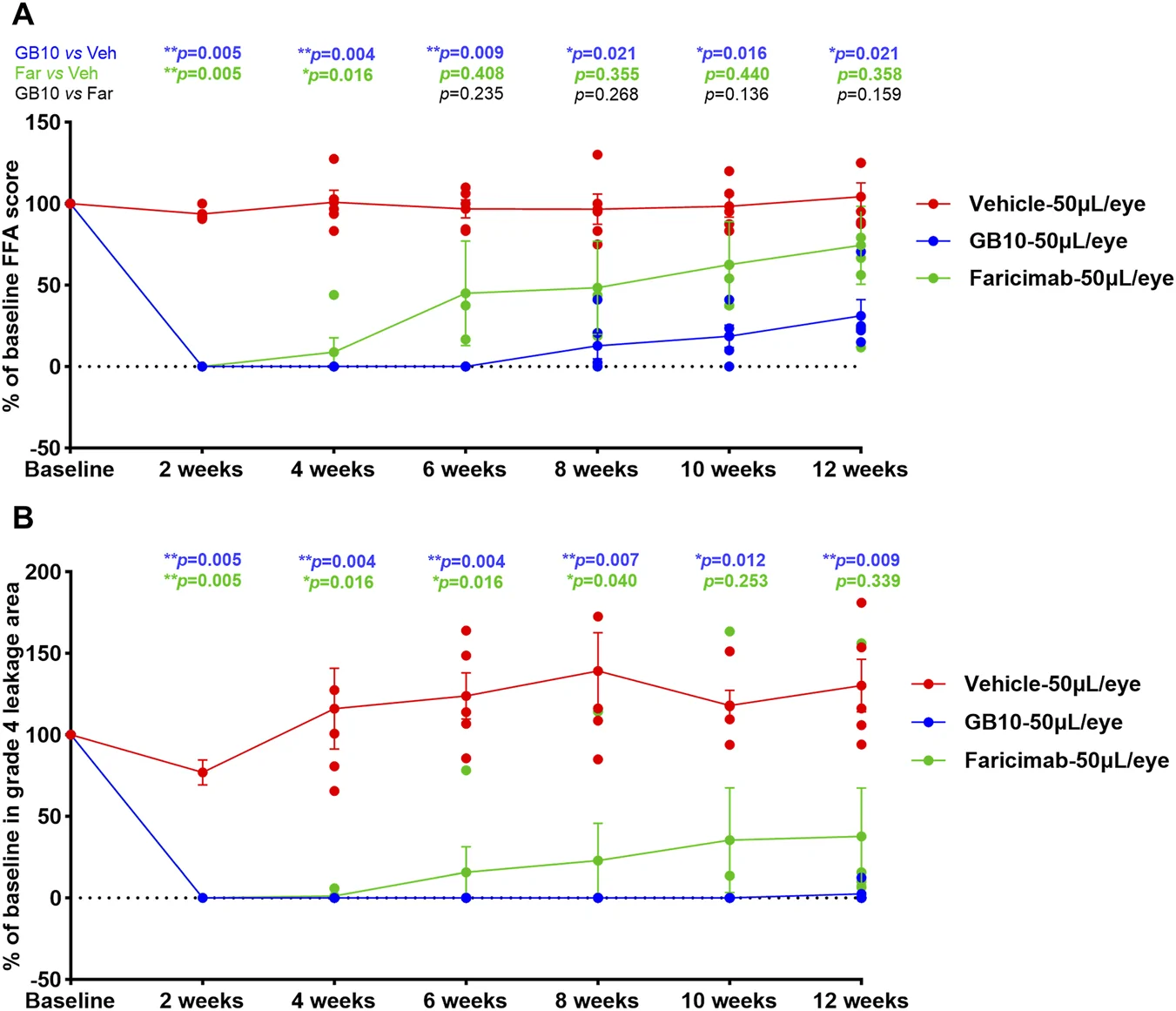
The changes of FFA score and grade 4 leakage area in rabbit RNV model. **(A)** The changes of FFA score. GB10 sustained improvement of FFA score through 12 weeks, whereas faricimab’s effect lasted for 4 weeks though short of statistical significance between GB10 and faricimab. **(B)** The changes of grade 4 leakage. GB10 sustained inhibition of grade 4 leakage area for 12 weeks, whereas faricimab’s effect on leakage area lasted until the week 8. *p < 0.05, **p < 0.01. Veh, vehicle; Far, faricimab. n = 5 eyes per group.

**TABLE 4:** Changes of FFA score (%) in rabbit RNV model.

Time point	Vehicle	GB10	Faricimab
Before administration	100.00 ± 0.00	100.00 ± 0.00	100.00 ± 0.00
2 weeks	93.71 ± 1.65	0.00 ± 0.00**	0.00 ± 0.00**
4 weeks	100.85 ± 7.37	0.00 ± 0.00**	8.82 ± 8.82*
6 weeks	96.79 ± 5.52	0.00 ± 0.00**	44.95 ± 32.16
8 weeks	96.71 ± 9.41	12.85 ± 8.06*	48.43 ± 28.46
10 weeks	98.46 ± 6.65	18.69 ± 6.91*	62.60 ± 26.34
12 weeks	104.33 ± 8.54	31.27 ± 9.98*	74.53 ± 23.94

Data were presented as Mean ± SEM; Compared with vehicle, *p < 0.05, **p < 0.01, ***p < 0.001. n = 5 eyes per group.

Since grade 4 leakage is associated with pathological neovascular leakage observed in patients, we evaluated therapeutic efficacy by quantifying the grade 4 leakage area. The percentage data of grade 4 leakage area at 2, 4, 6, 8, 10, and 12 weeks after administration compared with that before administration were detailed in Figure 5B and Table 5. The inhibition effects of GB10 and faricimab on grade 4 leakage area was similar with those observed for FFA scores. Compared with the vehicle, GB10 showed persistently reduced the retinal neovascularization leakage up to 12 weeks after administration. In contrast, faricimab displayed improvements only at 2–8 weeks, with little distinguishable change noted from week 10. In this preclinical rabbit RNV model, the observed temporal response indicate a numerically prolonged duration of anti-neovascular activity for GB10 relative to faricimab.

**TABLE 5:** Changes of grade 4 leakage area (%) in rabbit RNV model.

Time point	Vehicle	GB10	Faricimab
Before administration	100 ± 0.00	100 ± 0.00	100 ± 0.00
2 weeks	77.03 ± 7.68	0.00 ± 0.00**	0.00 ± 0.00**
4 weeks	116.12 ± 24.76	0.00 ± 0.00**	1.20 ± 1.20*
6 weeks	123.78 ± 14.27	0.00 ± 0.00**	15.64 ± 15.64*
8 weeks	139.12 ± 23.42	0.00 ± 0.00**	22.83 ± 22.83*
10 weeks	118.01 ± 9.38	0.00 ± 0.00*	35.41 ± 32.11
12 weeks	130.20 ± 16.14	2.48 ± 2.48**	37.74 ± 29.71

Data were presented as Mean ± SEM; Compared with vehicle, *p < 0.05, **p < 0.01. n = 5 eyes per group.

### 3.4 GB10 demonstrated high distribution in the target ocular tissues

After a single IVT of GB10 in chinchilla rabbits, distinct tissue distribution kinetics were observed. GB10 concentration in the retina and choroid peaked at 4h post-dose, whereas the time to maximum concentration (T_max_) was 168 h in aqueous humor, and 72 h in the cornea, lens, and serum. The terminal half-life (T_1/2_) for vitreous humor, retina, choroid, aqueous humor, lens, optic nerve and conjunctiva were 93.1 h, 94.2 h, 122 h, 37.8 h, 116 h, 101 h and 110 h, respectively (Table 6). The AUC_last_ in ocular tissues ranked in descending order as vitreous humor, retina, choroid, aqueous humor, cornea, lens, optic nerve, and conjunctiva (Figure 6). The exposure (AUC_last_) of GB10 in all ocular tissues were substantially greater than that in serum, with the AUC_last_ of ocular tissue to serum ranging from 14 to 4,787. The ocular distribution proportion in retina and choroid were 19.7% and 15.2%, respectively, accounting for approximately 35% of the total ocular distribution. This ocular tissue distribution pattern demonstrates preferential partitioning of GB10 to pathological target layers, providing pharmacokinetic rationale for the sustained anti-angiogenic efficacy observed in lesion grading assessments. In contrast, non-target tissues such as the optic nerve had much lower exposure than target tissues, and systemic exposure was minimal, with a serum exposure proportion of only 0.02% (Table 6). From these PK profile and ocular distribution, GB10 exhibited preferential partitioning into diseased target ocular tissues of retina and choroid, offering an explanation on GB10’s potency.

**TABLE 6:** PK Profile and Distribution of GB10 in chinchilla rabbits.

Tissue	AUC_last_ (h*μg/g)	C_max_ (μg/g)	T_max_ (h)	T_1/2_ (h)	AUC_last_, ocular to serum	Ocular distribution proportion
Vitreous humor^#^	517000	2,980	4.00	93.1	4787.00	NA
Retina	102000	590	4.00	94.2	944.00	19.70%
Choroid	78700	636	4.00	122	729.00	15.20%
Aqueous humor^#^	47900	192	168	37.8	444.00	9.30%
Cornea	22400	155	72.0	NA	207.00	4.30%
Lens	8,980	65.8	72.0	116	83.00	1.70%
Optic nerve	7,550	177	4.00	101	70.00	1.50%
Conjunctiva	1,510	36.9	4.00	110	14.00	0.30%
Serum^#^	108	0.914	72.0	NA	NA	0.02%

For the “AUC_last_, ocular to serum” and “Ocular distribution proportion” were rounded to two decimal places, all other parameters presented in the form of three significant figures. #, The unit of AUC_last_ of serum, vitreous humor, and aqueous humor was h*μg/mL, and the unit of C_max_ was μg/mL; NA, not available.

**FIGURE 6:**
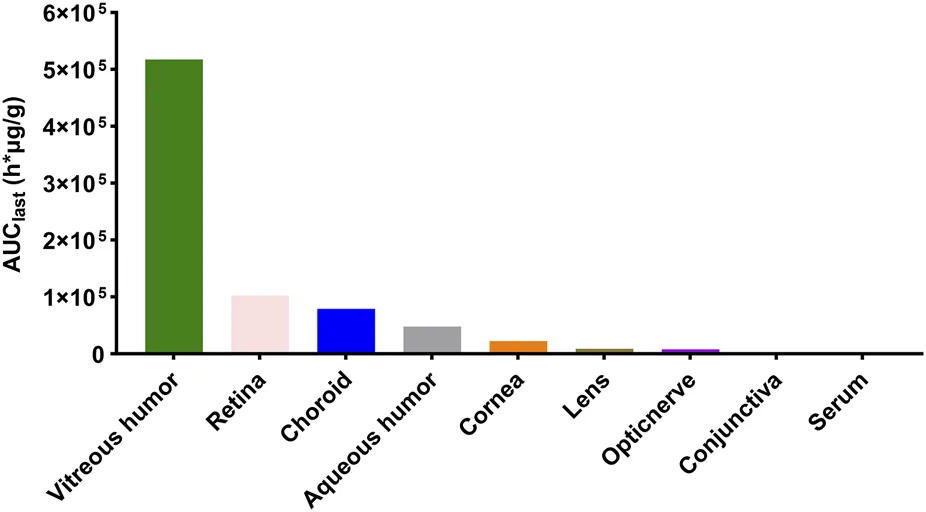
Ocular tissue distribution of GB10 in chinchilla rabbits. AUC_last_ of GB10 in chinchilla rabbits. GB10 could reach and be highly distributed in target tissues such as retina and choroid (neovascular lesions), and serum exposure was extremely low. The unit of AUC_last_ of serum, vitreous humor, and aqueous humor was h*μg/mL.

## 4 Discussion

Neovascular and exudative eye diseases such as nAMD, DME, DR and RVO are major causes of blindness globally, primarily affecting individuals aged 55 and older (Yau et al., 2012; Keenan et al., 2021; Rowe et al., 2025). The development of effective and durable therapies remains a pressing clinical challenge, despite the paradigm shift brought by anti-VEGF agents (Li et al., 2025). A major limitation of current treatments, such as bevacizumab, ranibizumab, and aflibercept is the frequent requirement for IVT injections, due to disease recurrence upon drug clearance, imposing substantial burden on patients and healthcare systems (Lukason et al., 2011). This highlights the urgent need for novel agents with a prolonged duration of action. The present study has characterized GB10, a novel bispecific VEGF/Ang-2 neutralizing fusion protein, for its efficacy and sustained inhibitory profile due to its tetravalent design and multitargeting.

This systematic dose-ranging study corroborated and extended our previous work (Wei et al., 2025), demonstrating numerically greater efficacy of GB10 versus faricimab in laser-induced cyno monkey CNV (n = 4 animal per group). At a dose of 0.7 mg/eye, the incidence of grade 4 leakage in the GB10 was 54.17%, lower than the 76.39% observed with the approved clinical dose of faricimab (6 mg/eye). Corresponding area of grade 4 leakage was 4.69 mm^2^ for GB10, compared with 6.73 mm^2^ for faricimab. This accelerated response at a lower dose of GB10 could be attributed to its multitargeting and molecular design. GB10 incorporated a VEGF-Trap domain at the N-terminus, enabling simultaneous high-affinity binding to VEGF-A, VEGF-B, and PlGF. This broad targeting capability may provide more comprehensive blockade of antigenic pathways compared with faricimab, which selectively binds VEGF-A. Structurally, GB10 adopted a fully symmetric, bivalent architecture, in contrast to the monovalent design of faricimab (Wei et al., 2025). This structural innovation is hypothesized to enhance target engagement efficiency and deepen signaling blockade, thereby providing a mechanistic basis for enhanced potency and durability. In fact, GB10 exhibited 7.5-fold lower IC_50_ for VEGF inhibition and 94-fold stronger inhibition of Ang-2/Tie-2 binding relative to faricimab. Key characteristics of GB10 and faricimab are summarized in Table 7. Collectively, these multi-dimensional advances support further translational investigation of GB10 as a next-generation bispecific fusion protein with neutralizing activity for clinical application.

**TABLE 7:** Comparative summary of structure, valence, target, efficacy between GB10 and faricimab.

Items	GB10	Faricimab	GB10 vs. Faricimab
Structure	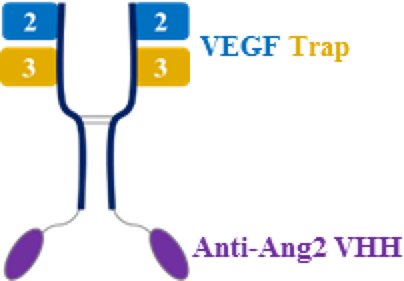	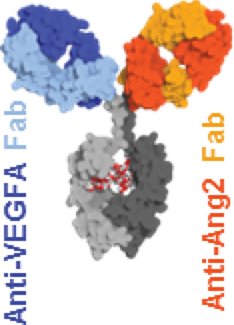	—
Molecular weight	127 kD	146 kD	—
Binding valence	2 + 2	1 + 1	Higher binding valence stoichiometry (2 + 2 vs*.* 1 + 1)
Target	VEGF-A VEGF-B PlGF Ang-2	VEGF-A Ang-2	Broader target coverage
VEGF inhibition (IC_50_)	0.02 nM	0.15 nM	7.5-Fold lower IC_50_ for VEGF inhibition
Ang-2 inhibition (IC_50_)	0.15 nM	14.17 nM	94-Fold lower IC_50_ for Ang-2 inhibition
Lowest effective dose in cyno CNV	0.7 mg/eye	6 mg/eye	Effective at a 9-fold lower dose
Earliest effective time point in cyno CNV	1 week	2 weeks	Earlier onset of efficacy (1 week vs. 2 weeks)
Maximal duration time point in rabbit RNV	12 weeks	4–8 weeks	Sustained efficacy through a longer observation window (12 weeks vs*.* 4–8 weeks)

Sustained efficacy assessment for long-lasting ophthalmic drugs requires preclinical models that recapitulate the chronic, non-regressing nature of human ocular neovascularization. Traditional models, such as laser-induced CNV, often exhibit spontaneous regression within weeks, limiting the assessment of sustained drug effects (Grossniklaus et al., 2010). We applied the DL-AAA-induced rabbit RNV model to evaluate duration of GB10, alongside faricimab. The RNV model develops measurable neovascular leakage as early as 4 weeks post DL-AAA injection; Leakage stabilizes between 4–8 weeks and persists for at least 24 weeks, consistent with previous characterization by Cao et al. (Cao et al., 2018). Within this model, a single IVT injection of GB10 brought clear reduction in RNV leakage within 2 weeks and maintained observable reduction in both FFA score and grade 4 leakage area at least for 12 weeks. In contrast, faricimab’s inhibitory effect on FFA score only lasted until the week 4, while its inhibitory effect on grade 4 leakage area persisted for 8 weeks. The extended duration of GB10 compares favorably to the previously reported 8-week efficacy of aflibercept in the same model (Cao et al., 2018). Mechanistically, GB10’s favorable preclinical activity may be attributed to its symmetric tetravalent architecture and multitargeting as demonstrated above. This potential of longer duration addresses a key unmet clinical need for reduced injection frequency (Boudousq et al., 2024; Rowe et al., 2025). It is worth to note that statistical comparisons between GB10 and faricimab revealed a difference that tended to grow across the extended observation period, but short of statistical significance due to small sample sizes and variability. Further verification with enlarged animal cohorts will be required to fully characterize and statistically compare the long-term anti-neovascularization leakage activity of the two agents.

The pharmacokinetic profile of GB10 provides additional mechanistic support for its development as a long-acting therapy for ocular neovascular diseases. GB10 rapidly distributed to key target tissues of retina and choroid with T_max_ of 4 h and persisted for an extended timeframe with T_1/2_ of 94.2 h and 122 h. Though no side to side comparison study with faricimab in this study, our previous work demonstrates that GB10 exhibits a longer vitreous half-life (59.4h) relative faricimab (39.6h) in New Zealand rabbit (Wei et al., 2025), which may contribute to sustained anti-leakage activity. Meanwhile GB10 displays delayed peak concentration in the aqueous humor (168 h post-dose), a pattern suggestive of redistribution from posterior segment tissues (del Amo et al., 2017; Parikh et al., 2017). Moreover, GB10 shows minimal systemic penetration, with a serum-to-vitreous AUC_last_ ratio of only 0.02%. Although this low systemic exposure represents a favorable pharmacokinetic characteristic (Li et al., 2012; Moisseiev et al., 2014), the available tissue concentration data remain insufficient to support definitive conclusions regarding local ocular or systemic safety.

This study highlighted several innovative aspects. First, GB10 at a lower dose (0.7 mg/eye) showed a trend toward more rapid therapeutic inhibition than the approved clinical dose of faricimab (6 mg/eye), with a rapid onset consistent with its ocular pharmacokinetics. More importantly, a trend of longer durability of GB10’s anti-leakage efficacy was validated in rabbit RNV model, overcoming key limitations of traditional transient CNV animal models. Furthermore, GB10 exhibited prominent retina/choroid distribution with a minimal serum exposure. Lastly, Ang2 VHH antibody has been applied for construction of bispecific fusion protein since single-domain antibodies represent promising molecular scaffolds for ocular biotherapeutics owing to its small molecular size, solubility, stability, penetration, and facile modular engineering (Jakobsen et al., 2026). Of course, its utility remains to be confirmed in clinical trials. Collectively, these preclinical observations identify GB10 as a promising candidate with potential to reduce treatment burden and improve patient adherence (Appell et al., 2024; Rakoczy, 2024).

Substantial limitations must be acknowledged to contextualize these preclinical results. First, although the DL-AAA induced rabbit model provided stable neovascular leakage, its pathology driven by gliotoxin-mediated neuronal degeneration does not fully replicate the complex, multifactorial etiology of clinical neovascularization (Goody et al., 2011; Cao et al., 2018). Second, formal PK/PD quantitative modeling linking tissue free-drug exposure magnitude to target occupancy and sustained lesion suppression was not implemented in this exploratory work, and future quantitative modeling analysis could strengthen the causal linkage between ocular distribution and prolonged anti-angiogenic activity. Third, FFA image analysis is not completely masked, which may introduce observer bias, despite pre-set objective scoring criteria and cross-evaluator arbitration procedures. Finally, GLP-compliant toxicology studies will be required for critical assessments of long-term safety. Thus, trials in patients will be necessary to validate whether the durable efficacy of GB10 observed can be translated into clinical benefits.

## 5 Conclusion

GB10, a novel bispecific VEGF/Ang-2 neutralizing fusion protein, displays promising preclinical potency and durable therapeutic effects in translational animal models of ocular neovascular diseases. In the cyno CNV model, GB10 achieved more rapid anti-leakage inhibition at a 9-fold lower dose relative to the clinical dose of faricimab (0.7 mg/eye vs. 6 mg/eye), showing numerically enhanced *in vivo* activity. Furthermore, in the rabbit RNV model, a single IVT of GB10 sustained suppression of grade 4 vascular leakage for over 12 weeks, longer than the 4–8-week efficacy for faricimab. These preclinical findings support GB10 as a promising candidate for further development as a long-acting therapy for ocular neovascular disorders; definitive clinical superiority remains to be validated through head-to-head clinical trial.

## Data Availability

The original contributions presented in the study are included in the article/supplementary material, further inquiries can be directed to the corresponding author.
